# Treatment differentiation between group music therapy and recreational choir singing for people with dementia and depression living in residential care homes: structured video analysis of the interventions in the MIDDEL trial

**DOI:** 10.3389/fpsyt.2025.1730949

**Published:** 2026-01-13

**Authors:** Jodie Bloska, Joanne Ablewhite, Naomi Rasing, Sarah Janus, Burçin Uçaner, Barış Gürkan, Christian Gold, Annemieke Vink, Justine Schneider

**Affiliations:** 1 Cambridge Institute for Music Therapy Research, Anglia Ruskin University, Cambridge, United Kingdom; 2 Institute of Mental Health, University of Nottingham, Nottingham, United Kingdom; 3 Department of Primary and Long-term Care, University of Groningen, University Medical Centre, Groningen, Netherlands; 4 Department of Musicology, Ankara Haci Bayram Veli University, Ankara, Türkiye; 5 Health & Social Sciences, NORCE Norwegian Research Centre, Bergen, Norway; 6 Music Therapy Department, ArtEZ University of the Arts, Academy of Music, Enschede, Netherlands

**Keywords:** choir singing, dementia, depression, music therapy, music-based interventions, residential care, treatment differentiation, video analysis

## Abstract

**Introduction:**

Music-based interventions are often implemented in residential dementia care to support quality of life and wellbeing, and to minimize neuropsychiatric symptoms such as depression. A recent international randomized controlled trial called MIDDEL (“Music Interventions for Dementia and Depression in Elderly Care”) compared efficacy of two popular music-based interventions: recreational choir singing (RCS) and group music therapy (GMT). The current study was undertaken to determine similarities and differences between the delivery of these two interventions within the MIDDEL trial.

**Methods:**

To determine treatment differentiation between RCS and GMT in MIDDEL, we undertook structured video analysis of a random sample of the available session videos from the trial. For each intervention, the videos were analyzed against a predefined checklist, which included items across three music-based activities: singing familiar songs, instrument playing and movement to music. Pearson’s chi-square tests were used to assess whether the type of intervention significantly influenced the delivery of the checklist items.

**Results:**

A sample of 65 session videos (GMT: 32; RCS: 33) were analyzed from MIDDEL sessions delivered in the Netherlands, Türkiye and the UK. Overall, the interventions differed significantly in their implementation, x^2^(1) = 101.39, *p* = <.001, with a small effect size (Cramer’s V = 0.257). Singing familiar songs was used in all GMT and RCS sessions, although there were some specific differences in terms of how facilitators engaged the participants within musical interactions. GMT sessions regularly incorporated playing instruments, whereas this was rare within RCS sessions. There was no difference in the use of movement to music, which occurred in around a third of sessions in both GMT and RCS.

**Discussion:**

The current findings confirm treatment differentiation between the two music-based interventions delivered as part of the MIDDEL trial. The results may indicate distinctive characteristics of professional music therapy as compared to community choir singing within residential dementia care, while also identifying overlaps in practice. Understanding the differences and similarities between these two popular music-based interventions can guide future research and inform their use in clinical practice.

## 1 Introduction

There are an estimated 57.4 million people living with dementia globally, with cases forecasted to rise to 152.8 million by 2050 (1). With limited effective curative treatments for dementia diseases (2), non-pharmacological psychosocial interventions continue to be essential to support quality of life and wellbeing for those affected. Depression is a common comorbid condition among those living with dementia (3) and can occur at any point throughout the course of the disease. It is common in the early stages of dementia (4) as well as upon admission to residential care (5), while recent research has also shown that higher cognitive impairment is associated with higher depressive symptoms (6). Clinical guidelines advise non-pharmacological psychosocial interventions as a first-line treatment for depression for those with dementia, with antidepressants only recommended for severe symptoms (7) due to adverse effects of psychotropic medications (8). Music-based interventions are a promising non-pharmacological approach to improve depressive symptoms based on potential biological, cognitive, social and emotional mechanisms of music engagement (9). A recent Cochrane review has indicated possible effectiveness of music-based interventions for depression in dementia (10). However, the evidence was found to be of low-to-moderate quality; one factor affecting research quality for music-based interventions is limited reporting of treatment fidelity within efficacy studies.

Treatment fidelity refers to whether an intervention has been delivered as intended within a research study. This is important within research investigating complex interventions, whereby several components may contribute to the effectiveness of an intervention. There are five elements of treatment fidelity: adherence to the intervention, dosage, quality of delivery, participant responsiveness, and treatment differentiation (11). Treatment differentiation involves identifying the unique and essential components required for the effectiveness of the intervention. Evaluating treatment differentiation within music-based interventions is significant considering the variety of ways music can be delivered and engaged in to potentially support health and wellbeing. Therefore, research to compare between different music-based interventions, techniques and approaches is necessary to determine unique components and has implications for future research and evidence-based practice.

Two popular music-based interventions often implemented in dementia care are choir singing and group music therapy. These interventions are both active music-based approaches, with distinct and overlapping components and potentially differing mechanisms and effects. These have recently been investigated in a randomized controlled trial called MIDDEL (“Music Interventions for Dementia and Depression in ELderly care”) (9, 12). Treatment fidelity and differentiation were carefully considered at the outset of the design of the interventions for the trial (13, 14), with protocolized manuals developed for delivery of both interventions. Treatment differentiation is important within this trial for interpreting differences between these two music-based interventions. Furthermore, although previous research has investigated outcomes of active versus receptive music therapy (15), there is a lack of literature that systematically differentiates between differing active approaches. The current study aims to confirm the treatment differentiation between the two active music-based interventions investigated in the MIDDEL trial, which also offers an opportunity to systematically investigate distinct and overlapping components between the implementation of choir singing and group music therapy for people living with dementia as delivered within this trial.

### 1.1 Study context: the MIDDEL trial

MIDDEL was an international randomized controlled trial that investigated the efficacy of group music interventions to reduce depressive symptoms among people with dementia living in care homes. The trial took place in Australia, the Netherlands, Norway, Türkiye, Germany and the UK from 1 May 2020 to 31 December 2023, with over 1000 residents participating across 86 care home units. Therefore, MIDDEL is the largest study to date of music-based interventions for people with dementia and depression residing in care homes, and the first to compare the effects of two different music-based interventions in this context – group music therapy (GMT) and recreational choir singing (RCS).

GMT and RCS differ in important ways, while also sharing some components. GMT emphasizes the therapeutic relationship between the music therapist and the person living with dementia, which is facilitated through active, reciprocal music making. This can include singing, playing instruments and movement to music, and makes use of both familiar music as well as musical improvisation. One principle of GMT is using musical elements to support affect regulation, where the music can help minimize distress, increase engagement or lift one’s mood. Another goal of GMT is to meet each individual’s psychosocial needs to support social and emotional wellbeing. Based on Kitwood’s (16, 17) theory of person-centered care, this also may reduce depressive symptoms and anxiety for people living with dementia. To meet individual needs, GMT is often carried out in smaller groups. Music therapists are allied health professionals, who have professional training and qualification in working with clinical populations as well as being skilled musicians. By comparison, RCS aims to encourage community and a familiar environment through singing together within a larger group. Sessions may focus on singing well-known songs, which are either personally or culturally relevant, or involve learning new material as a group. Choir leaders are skilled musicians with choir leading experience and skills, but do not require professional training or qualification to work with clinical populations.

The MIDDEL trial was designed as a pragmatic cluster-randomized trial with a 2x2 factorial design, where care home units were randomly allocated to one of four arms, receiving either: GMT sessions; RCS sessions; both GMT and RCS sessions; or usual care with no music intervention (the control group). The music intervention sessions were delivered over 6 months, where interventions were delivered twice weekly for 3 months and then once weekly during months 4-6. The primary aim of MIDDEL was to investigate the effectiveness of GMT, RCS, GMT in combination with RCS, or usual care in reducing depressive symptoms for care home residents with dementia. Secondary objectives were to examine the effects on cognitive function, neuropsychiatric symptoms, quality of life, dementia severity, functional impairment and mortality in the residents, in addition to workload factors affecting staff at the care home units. Outcomes were assessed at baseline, three months, six months and twelve months. Full details of the MIDDEL trial design and the interventions can be found in the published study protocol (9). The MIDDEL findings did not show any significant effects of the music interventions on the primary outcome of depressive symptoms or secondary resident outcomes at 6 months for the overall sample (12). There was a significant unfavorable effect of RCS on care staff burden. The effects contrasted across the six countries; in the overall sample for depressive symptoms, GMT consistently showed no significant effects across all countries, whilst RCS had positive effects in some countries (Australia, Norway and Türkiye) but had no effects or was harmful in others (Germany, the Netherlands and the UK) (12, 18, 19). Further subgroup analyses of the international sample revealed that the music interventions had a positive effect on depressive symptoms at 3 months for participants who attended at least 50% of sessions. Additionally, participants with moderate-to-severe dementia were more likely to experience positive effects of the music interventions for depressive symptoms than those with mild dementia. The current study compares the approaches and actual delivery of the two music-based interventions investigated in MIDDEL to provide context to the study’s main findings and to determine treatment differentiation within the trial. The comparison of these two interventions has implications for the use of music in dementia care more broadly, particularly for music practitioners in care settings, carers of people with dementia, researchers and all those who are concerned with increasing access to music in dementia care. The aim of this study was to characterize the two interventions used within the MIDDEL trial and to identify their similarities and differences in practice. The research question was therefore: *What were the similarities and differences in the delivery of GMT and RCS in the MIDDEL trial?*

## 2 Methods

This study presents a secondary analysis of intervention fidelity within the MIDDEL trial, with the aim to determine treatment differentiation by comparing the characteristics of two group music-based interventions for people living with dementia in residential care homes: GMT and RCS. Analysis of data collected as part of the MIDDEL trial was carried out using session videos recorded for intervention fidelity checking within the trial. GMT and RCS video analysis data were then compared statistically to determine similarities and differences in their delivery.

### 2.1 Interventions

Within the MIDDEL trial, residents residing in the care home units allocated to one of the intervention arms received group music sessions across 6-months. The first three months consisted of twice weekly sessions per intervention type, and the following three months consisted of once weekly sessions per intervention type. Each session was 45 minutes long. Music therapists and choir leaders were recruited and trained according to manuals for implementation developed as part of the trial. Music therapists were required to be qualified and registered with the appropriate professional association or registration body in their country. Choir leaders were required to be musicians with choir leading skills. In some countries, music therapists delivered both the GMT and RCS sessions following the intervention protocols. However, for clarity throughout this paper, GMT facilitators will be referred to as “music therapists” and RCS facilitators will be referred to as “choir leaders.” Both interventions were guided by specified fidelity checklists (see Supplementary Tables S1, S2) outlining the items interventionists were expected to deliver during each session, however, interventionists were also encouraged to use their professional judgement and flexibility in delivery was acceptable. Many intervention sessions took place despite the COVID-19 pandemic. Delivered sessions took place in-person, with no sessions being delivered either remotely or hybrid. Some sessions were impacted by COVID-19 restrictions, either being cancelled due to lockdowns or needing to be delivered within social distancing protocols and mask-wearing. While interventionists completed fidelity checklists after each session, they also made videos of themselves to provide a more objective insight into the process of the intervention delivery. The GMT manual and fidelity checklist were developed through a Delphi consensus with music therapists (20), and the RCS manual was developed through a systematic literature review (21).

### 2.2 Data collection

All intervention sessions delivered as part of the MIDDEL trial were audio-video recorded by the interventionists using tablets or laptops and automatically uploaded to a secure server at the University of Bergen in Norway. The session videos were made available to researchers undertaking video analysis as part of this study.

### 2.3 Video sample

This study utilized a convenience sample taken from the international MIDDEL research teams that were willing and had capacity to undertake the additional video analyses. This included three of the six participating countries: the Netherlands, Türkiye and the UK. The sample size of the videos was determined by the number of interventionists employed in each country involved. Three videos for each interventionist were sampled. For each interventionist, one session video was randomly selected from those delivered at each of three time-points across the six-month intervention period: early (months 1-2), middle (months 3-4) and late (months 5-6). Random selection was undertaken by using the random number generator function in Microsoft Excel. In cases where the randomly selected video was corrupt or incomplete, the video was excluded, and random selection was repeated to replace the video.

### 2.4 Video analysis

Video analysis was undertaken by raters from the research teams in each participating country and each randomly selected video was analyzed by a single rater. Videos were analyzed against a predefined checklist of session characteristics, which was the original GMT fidelity checklist for the MIDDEL trial. All items from the GMT fidelity checklist were utilized except for one that would not have been captured on video and was more relevant to the research process rather than the session delivery (“records attendance and reasons for non-attendance”). The GMT fidelity checklist was chosen because it included the core elements of both interventions as well as items that were felt to be potentially relevant to both interventions and could therefore potentially capture nuance between the two interventions. Raters watched the videos in real-time and rated each item as “done” or “not done”; an item was rated “done” if it occurred once or more and was rated “not done” if it did not occur at least once within the session.

### 2.5 Statistical analysis

To determine similarities and differences in session delivery between the two interventions, descriptive statistics and statistical tests of the video analysis checklists are reported and explored. Pearson’s chi-square tests (22–24), which assess the relationship between two categorical variables, were used to examine whether the type of intervention (GMT or RCS) impacted the session items that were delivered or not delivered and, therefore, whether there were significant differences in item delivery based on intervention type. They were conducted for each checklist item individually and for the total score. The chi-square statistics (x^2^) are reported alongside significance (chi-square and Fisher’s exact p-values) and effect size (Cramer’s V). The statistics were interpreted as either: (1) non-significant, interventions are very similar (p-value: >0.05); (2) small effect, slight difference between interventions (p-value: <0.05; Cramer’s V: 0.1-0.29); (3) medium effect, noticeable difference between interventions (p-value: <0.05; Cramer’s V: 0.3-49); or (4) large effect, substantial difference between the interventions (p-value: <0.001; Cramer’s V: >0.5).

### 2.6 Ethics

Ethical approval for the MIDDEL trial was obtained in each country from the appropriate human research ethics committee^1^. All participating residents provided formal written consent, either directly or by proxy or consultee (i.e., a next of kin or legal representative who provided advice indicating the person’s likely interest or desire in participating). This included agreeing to their sessions being video recorded, and the recordings being used for research purposes.

## 3 Results

**Figure 1:**
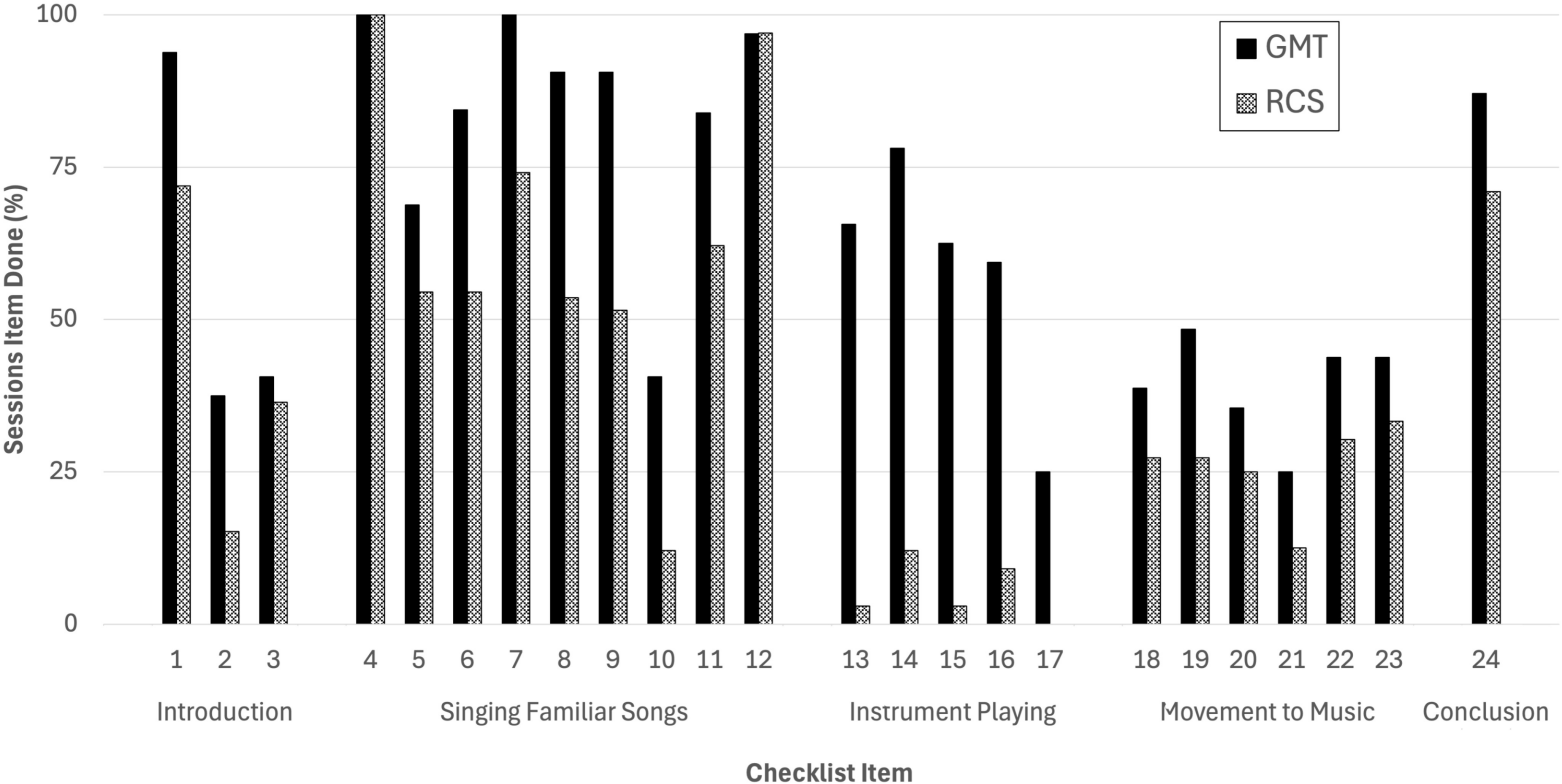
Completion rates of the video analysis checklist items for GMT and RCS sessions.

A total of 65 videos were analyzed from the Netherlands, Türkiye and the UK, which comprised 32 GMT videos and 33 RCS videos (see **Table 1**). **Table 2** presents the descriptive statistics for each item for both interventions alongside the chi-square test statistics. Overall, a chi-square test on total item scores revealed that the interventions differed significantly in their implementation, x^2^(1) = 101.39, *p* = <.001, with a small effect size (Cramer’s V = 0.257). The completion rates of the video analysis checklist items for GMT and RCS sessions are presented in a bar chart in **Figure 1**. Several noteworthy similarities and differences between the delivery of GMT and RCS were identified. These are explored below in detail for each of the main session activities indicated on the analysis checklist: (1) session introduction and conclusion, (2) singing familiar songs, (3) instrument playing and (4) movement to music.

**Table 1:** Sample of videos for analysis.

Country	GMT		RCS	
	Interventionists	Videos	Interventionists	Videos
Netherlands	8	20	6	15
Türkiye	1	3	2	6
UK	3	9	4	12
Total	12	32	12	33

**Table 2:** Video analysis checklist items with descriptive and Chi-square test statistics.

Item		Indicated on protocol		Sessions item done, N (%)		Chi-squared test results				Interpretation
		GMT	RCS	GMT	RCS	X^2^	df	chi-square p	Cramer’s V	
Session introduction:										
1	Facilitator uses consistent song to begin session (welcome song)	Yes	Yes	30 (93.8%)	23 (71.9%)	5.379	1	.020	.290	Small effect
2	Facilitator recaps previous sessions activities	Yes	Yes	12 (37.5%)	5 (15.2%)	4.201	1	.040	.254	Small effect
3	Facilitator outlines plans for the session	Yes	Yes	13 (40.6%)	12 (36.4%)	.125	1	.724	.044	Non-significant
Singing familiar songs:										
4	Facilitator engages participants in singing familiar/preferred songs	Yes	Yes	32 (100.0%)	33 (100.0%)	–	–	–	–	No variation
5	Facilitator facilitates song choice: moves from open- to close-ended choices as needed	Yes	No	22 (68.8%)	18 (54.5%)	1.385	1	.239	.146	Non-significant
6	Facilitator facilitates discussion/ reminiscence on at least one occasion in the session	Yes	No	27 (84.4%)	18 (54.5%)	6.786	1	.009	.323	Medium effect
7	Facilitator regularly engages each participant individually using eye contact, facial expression and gesture to encourage response	Yes	No	31 (100.0%)	20 (74.1%)	9.140	1	.003	.397	Medium effect
8	Facilitator acknowledges and mirrors or reflects participants’ spontaneous verbal and non-verbal responses (e.g. singing, vocalizing, moving)	Yes	No	29 (90.6%)	15 (53.6%)	10.484	1	.001	.418	Medium effect
9	Facilitator adapts music (extends songs where participants appear highly engaged, adapts tempo, volume, style to attune to overall group energy)	Yes	No	29 (90.6%)	17 (51.5%)	12.013	1	<.001	.430	Medium effect
10	Facilitator adapts music (as above) to encourage participation from participants displaying apathy or agitation	Yes	No	13 (40.6%)	4 (12.1%)	6.834	1	.009	.324	Medium effect
11	Facilitator uses appropriate facial expression/adapts gesture, and moves towards participants to encourage individuals and draw out responses	Yes	No	26 (83.9%)	18 (62.1%)	3.642	1	.056	.246	Non-significant
12	Facilitator uses a diversity of songs to meet musical interests/cultural background of the group	Yes	No	31 (96.9%)	32 (97.0%)	0.000	1	.982	.003	Non-significant
Instrument playing:										
13	Facilitator offers choices to participants of instruments to be played – first open-choices and if necessary then closed choices	Optional	No	21 (65.6%)	1 (3.0%)	28.429	1	<.001	.661	Large effect
14	Facilitator verbally and with gesture encourages participants to play along	Optional	No	25 (78.1%)	4 (12.1%)	28.643	1	<.001	.664	Large effect
15	Facilitator demonstrates how instruments are to be played and checks each participant knows how to play their instrument by asking participant to demonstrate the playing of the instrument.	Optional	No	20 (62.5%)	1 (3.0%)	26.272	1	<.001	.636	Large effect
16	Facilitator extends the duration of the song if participants are highly engaged in the performance of a song	Optional	No	19 (59.4%)	3 (9.1%)	18.346	1	<.001	.531	Large effect
17	If appropriate, facilitator encourages participants to play short solos on their instruments	Optional	No	8 (25.0%)	0 (0.0%)	9.408	1	.002	.380	Medium effect
Movement to music:										
18	Facilitator facilitates either spontaneous OR directed movement to music	Optional	No	12 (38.7%)	9 (27.3%)	.948	1	.330	.122	Non-significant
19	Facilitator models movements and encourages participants to move to the music both verbally/non-verbally	Optional	No	15 (48.4%)	9 (27.3%)	3.040	1	.081	.218	Non-significant
20	Spontaneous movement: Facilitator initiates/models movement to music and/or responds to/mirrors participants’ spontaneous movements to music	Optional	No	11 (35.5%)	8 (25.0%)	.822	1	.365	.114	Non-significant
21	Directed movement: Facilitator directs & models specific movements to music (e.g. dances associated with music/songs, specific exercises for head/neck, torso, arms, legs)	Optional	No	8 (25.0%)	4 (12.5%)	1.641	1	.200	.160	Non-significant
22	Movements are appropriate for participants’ physical abilities, interests and attention spans	Optional	No	14 (43.8%)	10 (30.3%)	1.261	1	.261	.139	Non-significant
23	Selected songs are upbeat in tempo and in keeping with participants’ musical preferences and physical abilities	Optional	No	14 (43.8%)	11 (33.3%)	.745	1	.388	.107	Non-significant
Session conclusion:										
24	Uses consistent song to conclude each session	Yes	Yes	27 (87.1%)	22 (71.0%)	2.433	1	.119	.198	Non-significant
Total				489 (64.2%)	297 (38.5%)	101.391	1	<.001	.257	Small effect

### 3.1 Session introduction and conclusion

The items on the checklist for the session introduction and conclusion included opening the session with a consistent welcome song, followed by a recap of the previous session and outline for that day’s session, and then closing the session with a consistent concluding song. These items were required as part of the delivery of both interventions. Both music therapists and choir leaders regularly used consistent welcome songs (Item 1) and concluding songs (Item 24); however, music therapists were slightly more likely to use a consistent welcome song than choir leaders (94% vs. 72%, *p* = .020).

Neither GMT nor RCS facilitators were very likely to recap the previous session (Item 2), although music therapists were also slightly more likely to do this (36% vs. 15%, *p* = .040). There was no difference between interventions as to whether the facilitator outlined that day’s session within the introduction (Item 3), with this not being implemented in the majority of sessions for both interventions. Previous MIDDEL findings have suggested that recapping and outlining the session were often deemed impractical by facilitators due to the memory impairments of the participants (25), which may explain the low implementation rate for these items. Overall, there were only slight differences in the delivery of the session introduction and conclusion between the interventions and, for both interventions, consistent welcome and concluding songs were often implemented.

### 3.2 Singing familiar songs

Singing familiar songs was a key activity for both GMT and RCS, with both interventions requiring this as part of the session delivery. Thus unsurprisingly, there was no variation between the interventions in the implementation of singing familiar or preferred songs (Item 4), with this being delivered in all the sessions analyzed. The GMT fidelity checklist further specified aspects for implementing singing of familiar songs, which did not appear on the RCS fidelity check. However, the results show that choir leaders just as regularly completed some of these specified items, including offering opportunities for song-choice from participants (Item 5), using facial expressions and movement towards participants to encourage responses (Item 11), and using a diversity of songs (Item 12). These items were not exclusive to the delivery of the GMT sessions, contrary to what was anticipated based on the protocolized interventions, which suggests these facilitator interactions are not unique to those with music therapy training. There were also several items that were noticeably carried out more frequently by music therapists, such as providing opportunities for discussions and reminiscence (Item 6) (84% vs. 55%, *p* = .009); engaging participants individually using eye contact, facial expressions and gesture (Item 7) (100% vs. 74%, *p* = .003); acknowledging and mirroring participants’ spontaneous verbal and non-verbal responses (Item 8) (91% vs. 54%, *p* = .001); and adapting or extending the music to attune to the group energy (Item 9) (91% vs. 52%, *p* = <.001) or support participants displaying apathy or agitation (Item 10) (41% vs. 12%, *p* = .009). Although these items appeared on the GMT checklist and not the RCS checklist, which means music therapists may have been more conscious to implementing them, the difference also aligns with music therapists’ training in recognizing and attuning to individuals’ non-verbal and musical responses. It may also have been more possible to implement these items within the smaller music therapy groups, as smaller participant numbers likely allow for more person-centered work. Overall, both GMT and RCS implemented singing familiar songs as a key activity of the sessions, but there were differences between how music therapists and choir leaders facilitated this group singing across specific components.

### 3.3 Instrument playing

The greatest differences in the implementation of GMT and RCS were found in the use of instruments, which was an optional activity for GMT and not included as a part of the RCS protocol. Although this activity was optional for music therapists, it was regularly implemented as part of the GMT sessions, whereas it was almost never initiated by choir leaders (66% vs. 3%, *p* = <.001). Music therapists consistently delivered most of the items related to instrument playing, such as offering a choice of instruments (Item 13), supporting and encouraging participants to play along (Items 14 and 15), and extending the duration of songs where participants are highly engaged (Item 16); the only item that was not regularly implemented by music therapists was encouraging participants to play short solos on their instruments (Item 17), which was only used within 25% of sessions. In general, instrument playing seems to have been a key activity within GMT sessions, although it was optional as part of the MIDDEL trial. Instrument playing was, unsurprisingly, rarely used in RCS sessions, which is likely due to the focus of these sessions on group singing and was not indicated on the RCS protocol. This could also be due to choir leaders having limited access to musical instruments while music therapists may more readily offer instruments as part of their regular practice.

### 3.4 Movement to music

Movement to music, which could be spontaneous or directed, was also included as an optional activity of GMT and not included as part of the RCS protocol. However, unlike instrument playing, there was no difference in the implementation of this activity between the GMT and RCS sessions, which both implemented movement to music in approximately one-third of sessions. Therefore, movement was used less frequently than instrument playing for music therapists, but more frequently than instrument playing for choir leaders. The use of movement to music by choir leaders is likely due to its accessibility, as movement to music can be a spontaneous and natural response when engaging in music activities and does not require any additional equipment. There were no significant differences between the interventions across any of the movement to music items (Items 18-20). For both GMT and RCS sessions, spontaneous movement to music (Item 20) was used slightly more than directed movement to music (Item 21).

## 4 Discussion

This paper presents findings from a secondary analysis of cross-national intervention fidelity data from the MIDDEL randomized controlled trial. The results confirm treatment differentiation between the two group music interventions for people with dementia that were investigated in the MIDDEL randomized controlled trial (GMT and RCS) and compared their delivery within the trial. The findings highlight overlaps between these two distinct music-based interventions, some of which were unexpected, while also confirming distinctive characteristics of professional music therapy as compared to community choir singing as delivered within the context of this research. Similar across both interventions was the use of singing familiar songs and movement to music, whereas playing instruments was a distinctive component of music therapy. Beyond looking at the activities used, completion of items related to the facilitators’ interaction behaviors reveal distinctions between how participants are engaged and supported within music therapy compared to choir sessions. Across both, facilitators offered song-choice, a diversity of songs and engaged participants through facial expressions and physically moving towards them. However, music therapists were more likely to encourage discussions and reminiscence, acknowledge or mirror participants’ verbal and non-verbal responses, adapt and extend the music based on group engagement, and support participants who were experiencing apathy or agitation.

Differences between the two interventions may stem from their underlying approaches and aims. Music therapy is informed by theories of personhood and person-centered care (16, 17), which may reflect the music therapists’ use of certain interactions to meet individual participants’ psychosocial needs. Music therapists are also trained in improvisation techniques, including mirroring, matching, extemporizing and frameworking (26), to adapt and extend the music based on participants’ verbal and non-verbal responses. This also includes employing the “iso principle” to support emotional regulation through adjusting musical elements to match and then gradually shift an individual’s mood (27, 28), which is often used to help minimize neuropsychiatric symptoms such as anxiety, apathy or depression. Therefore, music therapists’ training may have enabled them to adapt more sensitively to individual residents’ needs. This adaptive and flexible approach was emphasized in the protocolized GMT manual, and was confirmed in these results as well as anecdotally by session observers. Choir leaders also at times displayed these types of interactions and techniques, but significantly less than music therapists. This could reflect the success of the delivery of the interventions according to their protocols, especially as some choir leaders were also qualified music therapists. It could also reveal a difference in some choir leaders’ training or experience in working in care home settings with people with dementia, or the difference between the intervention approaches as choir sessions may follow a more group-centered rather than person-centered approach.

Overlaps and similarities between these group music interventions identified in the current results may also challenge the music therapy profession, as choir singing may offer a lower-cost alternative to specialized music therapy. However, there is a lack of qualified music therapists to meet the needs of every person living with dementia, especially given the increasing prevalence of dementia globally with an aging population (1, 29). This reflects previous findings that have questioned whether such approaches could deliver music therapy-like benefits without being limited by the availability of qualified therapists (30). If music therapy approaches are found to be more effective, tools like the CHORD manual (31), which was developed by music therapists to support dementia choir and singing group leaders, may be able to increase the benefits of other music-based interventions. While a manual cannot replace a master’s-level qualification, skill-sharing to support singing groups for people with dementia could potentially enable more facilitators to lead sessions that are “good enough” and increase access. Thus, it will be important for future research to identify if and how the approaches and qualification-level of music therapy compared to recreational choir singing might meet differing individual or community needs. This could ensure the limited access to specialized music therapy can be targeted to those with the greatest need.

The MIDDEL trial findings did not show any significant effects for either GMT or RCS on the primary outcome of depressive symptoms or the secondary resident outcomes of cognitive impairment, neuropsychiatric symptoms or quality of life (12). Subgroup analyses revealed that both music interventions may have more positive effects on depressive symptoms for those with moderate-to-severe dementia than for those with mild dementia. These findings suggest that the treatment differences between GMT and RCS generally did not lead to superior clinical outcomes for either intervention in the overall international sample. There was a significant unfavorable effect of RCS on care staff burden. It may be that care staff need to provide more support to choir leaders than music therapists to deliver sessions, perhaps due to the larger group sizes or choir leaders having less experience or training. Contrasting country outcomes suggest that cultural contexts may play a role in the effectiveness of music interventions. While GMT consistently showed no significant effects, RCS was found to be beneficial in some countries and ineffective or harmful in others. It may be that music therapists are more consistent in their delivery and standards across different countries, whilst choir leaders may be less so. There may also be differing responses to the components of RCS based on cultural norms.

### 4.1 Limitations

The limitations of this secondary analysis should be considered when interpreting the findings. First, fidelity was assessed using only the GMT fidelity checklist. While this tool provided a useful framework for comparison, it is important to note that several items were not indicated or required as part of the RCS sessions. Therefore, not all items were consistently applicable or expected across all facilitators, which may have led to discrepancies in fidelity ratings. For example, music therapists may have implemented certain techniques out of requirement based on inclusion the checklist, whereas choir leaders potentially did not perform these actions consciously based on exclusion from their checklist. This discrepancy raises questions about whether checklist items equally reflected the expectations across different facilitator types. Similarly, as the sessions were carried out to trial-specified protocols for research purposes, the “real-life” delivery of these interventions may differ in practice. This means that the differences and similarities found in the current study represent differences in the protocolized GMT and RCS sessions rather than differences in naturalistic delivery of music therapy and choir singing. However, the MIDDEL intervention protocols were developed through systematic explorations of current practice (20, 21) and aim to reflect current music therapy and choir singing delivery in residential dementia care. Second, fidelity coding was based on a binary (“yes” or “no”) coding analysis of item completion. While this approach provided a straightforward and replicable method for comparison, it may have oversimplified the complexity and nuance of facilitator delivery. A more detailed rating system (e.g., Likert scales or qualitative observation) could provide deeper insight into the quality and style of implementation. Due resource limitations, coding was conducted by single raters, meaning that inter-rater reliability was not assessed and evaluations of interventionists’ behaviors may be inconsistent. Third, the fidelity analysis was limited to data from three of the six participating countries of the MIDDEL trial. This partial coverage may limit the generalizability of the findings, especially in terms of cross-cultural variations in program delivery and interpretation of fidelity measures. Finally, program delivery occurred during the COVID-19 pandemic, which may have constrained facilitators’ ability to fully enact certain checklist items. For example, restrictions on physical proximity and facial visibility (e.g., due to mask-wearing or remote delivery) may have hindered the use of gestures, facial expressions, and physical movement toward participants, which are elements that are often integral to effective group music facilitation.

### 4 Conclusion

This paper closely examined and compared the delivery of music therapy and choir singing for people with dementia and depression residing in care homes within a large international randomized controlled trial called MIDDEL. It used data from three countries – the Netherlands, Türkiye and the UK – to compare music interventions that to a casual observer might appear identical and confirm treatment differentiation within the MIDDEL trial. With the growing awareness of the benefits of music for people with dementia, it is important to specify what we mean by music-based interventions and differentiate between the delivery, aims and benefits of different uses of music. This study confirms that, within the MIDDEL trial, the delivery of music therapy by qualified professionals is different in identifiable ways from the delivery of informal choral singing. Further research is necessary to address whether differences in input are reflected in outcomes for participants and, if so, who benefits in what ways from which intervention.

## Data Availability

The audio-video footage used for the video analysis is not available by request due being identifiable data. The raw dataset from the coded video analysis supporting the conclusions of this article will be made available by the authors, without undue reservation. Requests to access the datasets should be directed to jodie.bloska@aru.ac.uk.

## Footnotes

1 Netherlands: Medical Ethics Review Board (METc) of the University Medical Center Groningen (reference: UMCG 202000194); Türkiye: Ethics commission at the Ankara Haci Bayram Veli University (reference: 2021/100); UK: Health Research Authority (Wales Research Ethics Committee) (reference: 20/WA/0226).
