# Supplementary material for: Treatment differentiation between group music therapy and recreational choir singing for people with dementia and depression living in residential care homes: structured video analysis of the interventions in the MIDDEL trial
Source: Front Psychiatry. 2026 Jan 13;16:1730949. doi: 10.3389/fpsyt.2025.1730949 (PMC12835769; doi:10.3389/fpsyt.2025.1730949)
Supplement: Supplementary file 1 [file Table1.docx]

Group Music Therapy (GMT) Fidelity Checklist

| Session Introduction (5 minutes): | Yes   No |
| --- | --- |
| MT uses consistent song to begin session | Done  Not Done |
| MT recaps previous sessions activities | Done  Not Done |
| MT outlines plans for the session | Done  Not Done |
| MT records attendance and reason for non-attendance | Done  Not Done |
| Activity 1: Singing familiar songs (15 minutes) | Yes   No |
| MT engages participants in singing familiar/preferred songs | Done  Not Done |
| MT facilitates song choice: moves from open- to close-ended choices as needed | Done  Not Done |
| MT facilitates discussion/ reminiscence on at least one occasion in the session | Done  Not Done |
| MT regularly engages each participant individually using eye contact, facial expression and gesture to encourage response | Done  Not Done |
| MT acknowledges and mirrors or reflects participants’ spontaneous verbal and non-verbal responses (e.g. singing, vocalizing, moving) | Done  Not Done |
| MT adapts music (extends songs where participants appear highly engaged, adapts tempo, volume, style to attune to overall group energy) | Done  Not Done |
| MT adapts music (as above) to encourage participation from participants displaying apathy or agitation | Done  Not Done  N/A |
| MT uses appropriate facial expression/adapts gesture, and moves towards participants to encourage individuals and draw out responses | Done  Not Done |
| MT uses a diversity of songs to meet musical interests/cultural background of the group | Done  Not Done |
| Activity 2: Instrument playing (5-7 minutes) - OPTIONAL | Yes   No |
| MT offers choices to participants of instruments to be played – first open-choices and if necessary then closed choices | Done  Not Done |
| MT demonstrates how instruments are to be played and checks each participant knows how to play their instrument by asking participant to demonstrate the playing of the instrument. | Done  Not Done |
| MT verbally and with gesture encourages participants to play along | Done  Not Done |
| MT extends the duration of the song if participants are highly engaged in the performance of a song | Done  Not Done |
| If appropriate, MT encourages participants to play short solos on their instruments | Done  Not Done |
| Activity 3: Spontaneous or directed movement to music (5-7 minutes) - OPTIONAL | Yes   No |
| MT facilitates either spontaneous OR directed movement to music | Done  Not Done |
| MT models movements and encourages participants to move to the music both verbally/non-verbally | Done  Not Done |
| MT spontaneous movement: initiates/models movement to music and/or responds to/mirrors participants’ spontaneous movements to music | Done  Not Done |
| Directed movement: MT directs & models specific movements to music (e.g. dances associated with music/songs, specific exercises for head/neck, torso, arms, legs) | Done  Not Done |
| Movements are appropriate for participants’ physical abilities, interests and attention spans | Done  Not Done |
| Selected songs are upbeat in tempo and in keeping with participants’ musical preferences and physical abilities | Done  Not Done |
| Activity 4: Singing familiar songs (6-10 minutes) – See Activity 1 | Yes   No |
| Session Closure: | Yes   No |
| Uses consistent song to conclude each session | Done  Not Done |
