# Supplementary material for: Treatment differentiation between group music therapy and recreational choir singing for people with dementia and depression living in residential care homes: structured video analysis of the interventions in the MIDDEL trial
Source: Front Psychiatry. 2026 Jan 13;16:1730949. doi: 10.3389/fpsyt.2025.1730949 (PMC12835769; doi:10.3389/fpsyt.2025.1730949)
Supplement: Supplementary file 2 [file Table2.docx]

Recreational Choral Singing (RCS) Fidelity Checklist

| Session Introduction: | Yes   No |
| --- | --- |
| Facilitator uses consistent song to begin session (welcome song) | Done  Not Done |
| Facilitator recaps previous sessions activities | Done  Not Done |
| Facilitator outlines plans for the session | Done  Not Done |
| Facilitator records attendance and reason for non-attendance (eg. Sick) | Done  Not Done |
| Session Activities: | Yes   No |
| Facilitator provides simple physical warm up: breathing, stretching, posture awareness (3-4 mins) | Done  Not Done |
| Facilitator provides simple vocal warm up: breathing, stretching, posture awareness: vocal agility exercises, dynamics, harmony, humming/vowel prolongations, scales/glides/diction, arpeggios and 3rds scales (3-4 mins) | Done  Not Done |
| Facilitator engages participants in singing participant-selected songs for 30 mins | Done  Not Done |
| Songs are performed with comfortable range  range | Done  Not Done |
| Facilitator provides lyrics in large print | Done  Not Done |
| Session Closure: | Yes   No |
| Facilitator used consistent song to conclude each session | Done  Not Done |
